# Overexpression Analysis of *PtrLBD41* Suggests Its Involvement in Salt Tolerance and Flavonoid Pathway in *Populus trichocarpa*

**DOI:** 10.3390/ijms252212349

**Published:** 2024-11-17

**Authors:** Jiewan Wang, Yi Liu, Xingshun Song

**Affiliations:** 1 Key Laboratory of Saline-Alkali Vegetation Ecology Restoration (Northeast Forestry University), Ministry of Education, Harbin 150040, China; jiewan_wang@163.com (J.W.); liuyi1070156066@163.com (Y.L.); 2 College of Life Science, Northeast Forestry University, Harbin 150040, China

**Keywords:** *Populus trichocarpa*, transcriptome, LBD family, transcription factor, *PtrLBD41*, salt stress, flavonoid

## Abstract

Soil salinization is a significant environmental stress factor, threatening global agricultural yield and ecological security. Plants must effectively cope with the adverse effects of salt stress on survival and successful reproduction. Lateral Organ Boundaries (LOB) Domain (LBD) genes, a gene family encoding plant-specific transcription factors (TFs), play important roles in plant growth and development. Here, we identified and functionally characterized the LBD family TF PtrLBD41 from *Populus trichocarpa*, which can be induced by various abiotic stresses, including salt, dehydration, low temperature, and Abscisic Acid (ABA). Meanwhile, transgenic plants overexpressing *PtrLBD41* showed a better phenotype and higher tolerance than the wild-type (WT) plants under salt stress treatment. Transcriptome analysis found that the differentially expressed genes (DEGs) between the WT and overexpression (OE) line were enriched in the flavonoid biosynthetic process, in which chalcone synthases (CHS), naringenin 3-dioxygenase (F3H), and chalcone isomerase (CHI) were significantly up-regulated under salt stress conditions through qRT-PCR analysis. Therefore, we demonstrate that PtrLBD41 plays an important role in the tolerance to salt stress in *P. trichocarpa*.

## 1. Introduction

Abiotic environmental factors, including salinity, are major plant stressors that can limit plant development and productivity, resulting in agricultural yield losses with significant economic consequences for growers [1,2,3,4]. High-salinity stress usually refers to the high-osmotic-potential environment caused by the ion chloride (Cl^−^) and sodium (Na) in the soil [5]. The accumulation of salt in soil can produce osmotic pressure, which impedes the absorption of water and the opening and closing of stomata, hence inhibiting the normal growth of plants [6,7]. Exposure to a high-salinity environment affects the photosynthesis of plants and disrupts chloroplast synthesis, causing plant dwarfism, leaf yellowing, and a reduced root length [8]. Salt stress affects water uptake and produces ionic toxic effects, leading to disrupted ion homeostasis and various metabolic disorders, while contributing to the increase in reactive oxygen species (ROS) concentration [9,10,11].

The Lateral Organ Boundaries Domain (LBD) family proteins or ASYMMETRIC LEAVES2-LIKE (ASL) proteins constitute plant-specific transcription factors (TFs) with conserved Lateral Organ Boundaries (LOB) domains [12]. LBD TFs contain three specific motifs, CX_2_CX_6_CX_3_C, GAS-block, and LX_6_LX_3_LX_6_L, which are divided into classes I and II based on the structure [13,14]. Most members of class I contain one zinc finger motif CX_2_CX_6_CX_3_C, one GAS-block motif, and one LX_6_LX_3_LX_6_L spiral coiled structure [15,16], while members of class II only contain one motif similar to the zinc finger CX_2_CX_6_CX_3_C [17]. A large number of studies have shown that LBD TFs are involved in the development of plant leaves, lateral roots, inflorescence, and embryos, as well as the regulation of secondary growth [18,19,20,21,22,23,24].

*Populus trichocarpa*, a perennial woody plant whose genome sequencing was completed and published in 2006, is widely used as a model plant for physiological and genetic studies [25]. In poplars, *PtaLBD1/4/15/18* was predominantly expressed in wood-forming tissues [23]. The overexpression of *PagLBD3* increased stem secondary growth in *Populus* with a significantly higher rate of cambial cell differentiation into phloem [26]. Mutations in *PtrLBD39* and *PtrLBD22* of *P. trichocarpa* are observed to have resulted in increased lignin content and decreased cellulose content in tension wood [27]. *PtrLBD39* functions as a repressor, regulating both primary and secondary growth [28]. As for abiotic stress, although current studies on LBDs remain limited, it is evident that the overexpression of *PvLBD12* enhances salt tolerance by altering a wide range of physiological responses in *Panicum virgatum L.*, such as increased proline accumulation, reduced malondialdehyde (MDA) production, improved potassium ion (K+) accumulation, and reduced Na+ absorption [29]. When plants were subjected to salt stress, the constitutive expression of *MtLBD1* roots changed their overall structure in *Medicago truncatula* [30]. The overexpression of *PheLBD29* in *Arabidopsis* increased the tolerance to drought stress by accumulating more soluble sugars and less MDA [31]. *ZmLBD5* from *Zea mays* regulates drought tolerance by impairing abscisic acid [32] and suppressing ROS accumulation [33]. The *ZmLBD2* gene positively regulates Hydrogen peroxide (H_2_O_2_) homeostasis in plants to enhance plant drought resistance [34]. However, studies on the function of *P. trichocarpa* LBD TFs under salt stress remains sparse by far.

In our study, we have isolated and cloned an LBD TF family gene *PtrLBD41*. The experimental results illustrated that *PtrLBD41* is induced by abiotic stress and the overexpression of *PtrLBD41* can enhance the salt tolerance of transgenic *P. trichocarpa*. This research aims to provide a theoretical framework for further study of the functions and molecular regulatory mechanisms of *PtrLBD41*.

## 2. Results

### 2.1. Cloning and Bioinformatic Analysis of PtrLBD41

**Figure 1:**
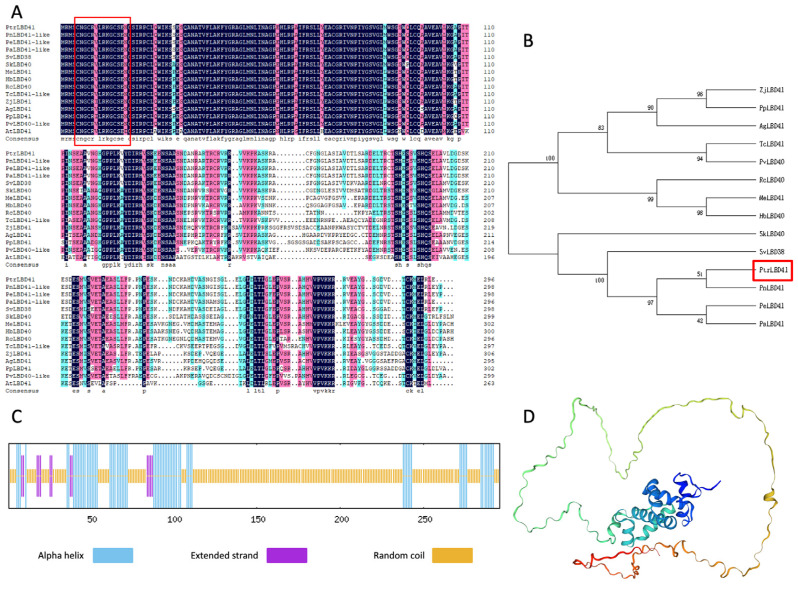
Contrast and evolutionary relationship between PtrLBD41 and LOB domain-containing proteins in different species, and prediction of PtrLBD41 protein domains and structure. (**A**) Comparison between homology of PtrLBD41 protein and LOB domain-containing proteins in other plants, with conserved amino acids shaded in different colors. The conserved regions of the amino acid sequence are marked by black and red boxes. The accession numbers are as follows: PnLBD41-like (XP_061947259.1, *Populus nigra*), PeLBD41-like (XP_011021242.1, *Populus euphratica*), PaLBD41-like (XP_034889549.1, *Populus alba*), SvLBD38 (KAJ6684372.1, *Salix viminalis*), SkLBD40 (KAJ6738787.1, *Salix koriyanagi*), MeLBD41 (XP_021602536.1, *Manihot esculenta*), HbLBD40 (XP_021643936.1, *Hevea brasiliensis*), RcLBD40 (XP_048231049.1, *Ricinus communis*), TcLBD41-like (XP_017975845.1, *Theobroma cacao*), ZjLBD41 (XP_015880531.3, *Ziziphus jujuba*), AgLBD41 (XP_062150573.1, *Alnus glutinosa*), PpLBD41 (XP_007215745.1, *Prunus persica*), PvLBD40-like (XP_031250561.1, *Pistacia vera*), and AtLBD41 (NP_566175.1, *Arabidopsis thaliana*). Red block: The CX2CX6CX3C zinc finger-like domain. (**B**) Phylogenetic tree analysis of NAC protein in *Fragaria vesca* and other plants. The red frame is the target protein. (**C**) Predicted protein secondary structure of PtrLBD41 protein using the SPOMA. (**D**) Tertiary structure of PtrLBD41 protein predicted by Expasy.

The LBD TF PtrLBD41 (XP_006383458.1) was isolated from *P. trichocarpa* (Figure S1), and the sequencing results are shown in Figure 1A. The full length of *PtrLBD41* is 891 bp, and the results of the ExPASy-ProtParam analysis predicted that the PtrLBD41 protein consists of 297 amino acids, of which Ser (10.8%), Ala (9.8%), Leu (9.1%), and Val (8.1%) are of the highest proportions. PtrLBD41 is a hydrophilic protein with a −0.270 grand average of hydropathicity (GRAVY).

Sequence analysis revealed that the PtrLBD41 protein belongs to the LBD TF family, with an LOB conserved domain but no obvious transmembrane structure. Our research constructed a phylogenetic tree to investigate the affinities of PtrLBD41 by comparing the amino acid sequences of PtrLBD41 protein with the sequences of LOB domain-containing proteins from other species. PtrLBD41 was determined to possess a similarity with *Populus nigra* PnLBD41 (XP_061947259.1), *Populus euphratica* PeLBD41 (XP_011021242.1), and *Populus alba* PaLBD41 (XP_034889549.1) with a high homology (Figure 1B). The predicted secondary structure of the encoded proteins using SOPMA showed that the secondary structure of the PtrLBD41 was composed of 24.32% α-helix, 70.61% random coil, and 5.07% extended strand, with no evidence of β-turn (Figure 1C,D).

### 2.2. Subcellular Localization of PtrLBD41

**Figure 2:**
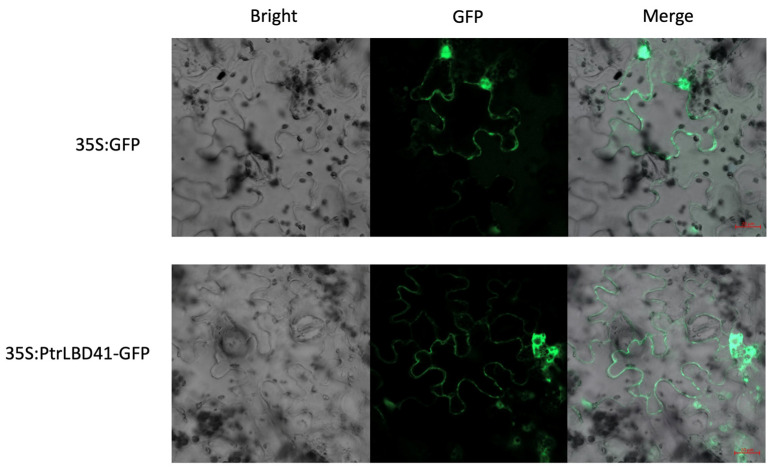
Subcellular localization of PtrLBD41 protein in tobacco leaf lower epidermal cells, based on visualization of green fluorescent protein (GFP) in tobacco leaves transformed with a fusion construct (35S: PtrLBD41-GFP) or empty vector (35S: GFP). Bright-field images, GFP fluorescence, and merged images are displayed from left to right. Fluorescence was observed by confocal microscopy. Scale bar is 20 μm.

To determine the subcellular localization of PtrLBD41, a 35S::PtrLBD41-GFP (Green fluorescent protein) vector was constructed by fusing a GFP tag. Micrographs showed that cells transformed with the PtrLBD41-GFP vector exhibits GFP fluorescence signals in the cell membrane specifically, whereas the control GFP protein was found to be present throughout the entire cell (Figure 2). Therefore, it can be safely stated that PtrLBD41 is localized to the cell membrane.

### 2.3. Expression Analysis of PtrLBD41 upon Different Abiotic Stresses

**Figure 3:**
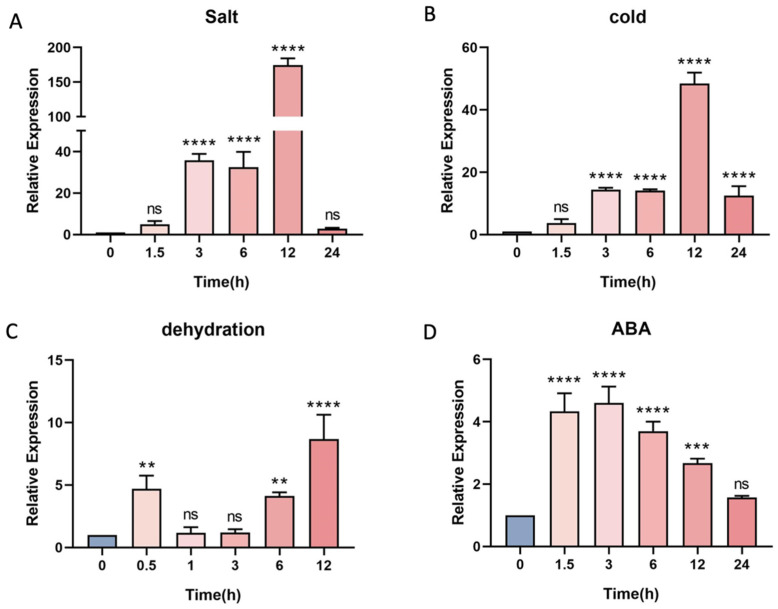
Expression pattern analysis of *PtrLBD41* in *Populus trichocarpa* under abiotic stress by qRT-PCR. Time-course of *PtrLBD41* expression in young leaf in the control and under (**A**) salt (200 mM NaCl), (**B**) low-temperature (4 °C), (**C**) dehydration, and (**D**) abscisic acid (100 μM ABA) treatments. Significant differences are marked with asterisks above the error bar (Student’s *t*-test; ** *p* ≤ 0.01, *** *p* ≤ 0.001, **** *p* < 0.0001, ns: not significant).

To assess the impact of abiotic stresses on *PtrLBD41* expression, we analyzed the gene expression using a quantitative real-time polymerase chain reaction (qRT-PCR). Evidently, *PtrLBD41* is sensitive to all these treatments, among which the salt treatment resulted in the highest up-regulation (Figure 3A). The expression of *PtrLBD41* began to be up-regulated at 3 h and reached the peak level at 12 h under salt and cold stress (Figure 3A,B). The dehydration of the plant leaves immediately caused the *PtrLBD41* expression to up-regulate before a visible decrease, followed by a second increase at 6 h (Figure 3C). After Abscisic Acid (ABA) treatment, an up-regulation of *PtrLBD41* expression was immediately observed which has lasted for 12 h (Figure 3D). In summary, *PtrLBD41* can be significantly induced by salt, cold, dehydration, and ABA stresses.

### 2.4. Overexpression of PtrLBD41 Enhances the Tolerance of P. trichocarpa to Salt Stress

**Figure 4:**
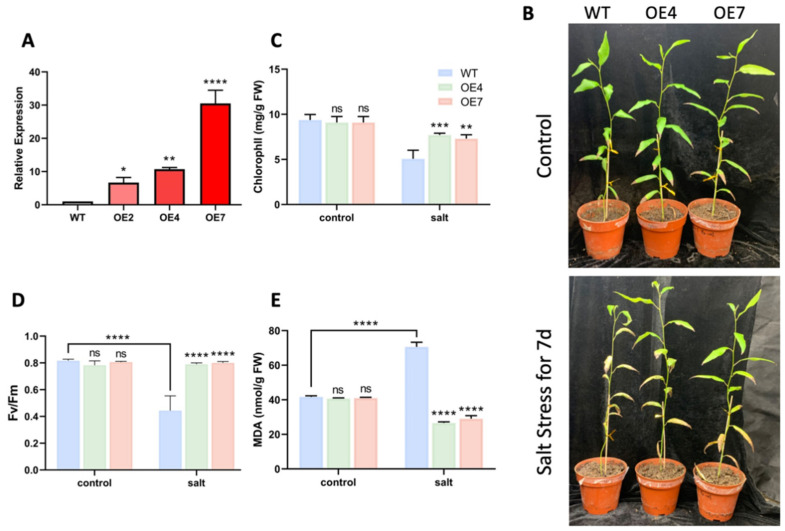
Salt treatment of transgenic *P. trichocarpa* lines overexpressing *PtrLBD41*. (**A**) Relative expression level of *PtrLBD41* in WT, and overexpression lines (OE2, OE4, and OE7). (**B**) Phenotypes of the WT and transgenic lines (OE4 and OE7) grown in the control environment and salt treatment (irrigation with 200 mM NaCl for 7 days). Chlorophyll contents (**C**), Fv/Fm (**D**), and MDA contents (**E**) in the WT and transgenic lines (OE4 and OE7) under NaCl treatment for 7 days. Significant differences are marked with asterisks above the error bar (Student’s *t*-test; * *p* ≤ 0.05, ** *p* ≤ 0.01, *** *p* ≤ 0.001, **** *p* < 0.0001, ns: not significant).

To reveal the role of *PtrLBD41* in salt tolerance in *P. trichocarpa*, we constructed a number of transgenic *P. trichocarpa* lines with the overexpression of *PtrLBD41.* A total of three lines (Line2, Line4, and Line7) of *PtrLBD41* overexpression lines (OEs) were thus obtained with an increased expression level in OE lines in comparison with control plants (Figure 4A). OE4 and OE7 plants with a higher transcript abundance were selected for further study (Figure 4A). In the absence of any stress treatment, the development of each line was essentially the same. Then, the wild-type (WT), OE4, and OE7 were subsequently treated with an NaCl solution of 200 mM for seven days. Phenotypic observations showed that the WT plants were severely affected under NaCl treatment, showing wilting and yellowing, compared to overexpressing plants (Figure 4B). In addition, some physiological indices reflecting the stress degree of plants were measured: the Fv/Fm (optimal/maximal photochemical efficiency of PSⅡ in the dark) level and chlorophyll content of the overexpressing plants were found to be significantly higher, while the MDA levels were significantly lower than that of the WT plants after the salty treatment (Figure 4C–E). The above results indicated that the overexpression of *PtrLBD41* in *P. trichocarpa* could alleviate the damage caused by salt stress. To sum up, our results suggest that *PtrLBD41* functions as a positive regulator in the response to salt stress in *P. trichocarpa*.

### 2.5. Transcriptome Analysis of PtrLBD41 Transgenic Plants and WT Plants

**Figure 5:**
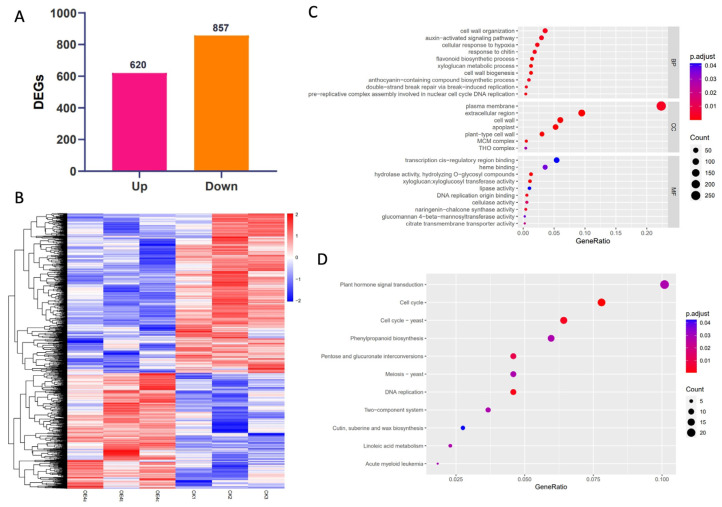
Transcriptome analysis of *PtrLBD41* transgenic plants and WT plants. (**A**) Histogram analysis of the number of DEGs of WT and *PtrLBD41*-OE plants. (**B**) Heat map of DEGs. (**C**) GO analysis of DEGs. The vertical coordinate indicates GO entries; the horizontal coordinate indicates the ratio of the number of genes enriched in the entry to the total number of genes; the color indicates *p* adjust, the redder the color, the higher the significance; the size of the bubble indicates the number of genes enriched in the entry, a bigger bubble indicates more genes. (**D**) KEGG analysis of DEGs. The vertical coordinate indicates the name of the pathway; the horizontal coordinate indicates the proportion of the number of genes enriched in the pathway to the total number of genes; the color indicates p-adjust, the redder the color, the higher the significance; the size of the bubbles indicates the number of genes enriched in the pathway, bigger bubbles indicate more genes. MF: molecular function, CC: cellular component, BP: biological process.

Next, we performed a comparative transcriptome analysis using leaves from plants of the *PtrLBD41*-OE4 lines and WT plants. The RNA-seq (Ribonucleic Acid sequencing) data obtained showed that 620 genes have been up-regulated and 857 genes down-regulated in PtrLBD41-OE as compared with the WT (fold change ≥ 2, Figure 5A,B). Moreover, GO (Gene Ontology) and KEGG (Kyoto Encyclopedia of Genes and Genomes) enrichment analyses were also performed in order to determine which processes have been affected. The GO enrichment analysis showed that the differential expressed genes (DEGs) were enriched in the plasma membrane (GO:0005886) and extracellular region (GO:0005576) in terms of the cellular component; the cell wall organization (GO:0071555), auxin-activated signaling pathway (GO:0009734), and flavonoid biosynthetic process (GO:0009813) in terms of the biological process; and transcription cis-regulatory region binding (GO:0000976) in terms of molecular function (Figure 5C). The KEGG enrichment analysis showed that the DEGs were enriched in plant hormone signal transduction (map04075), cell cycle (map04110), phenylpropanoid biosynthesis (map00940), etc. (Figure 5D).

**Figure 6:**
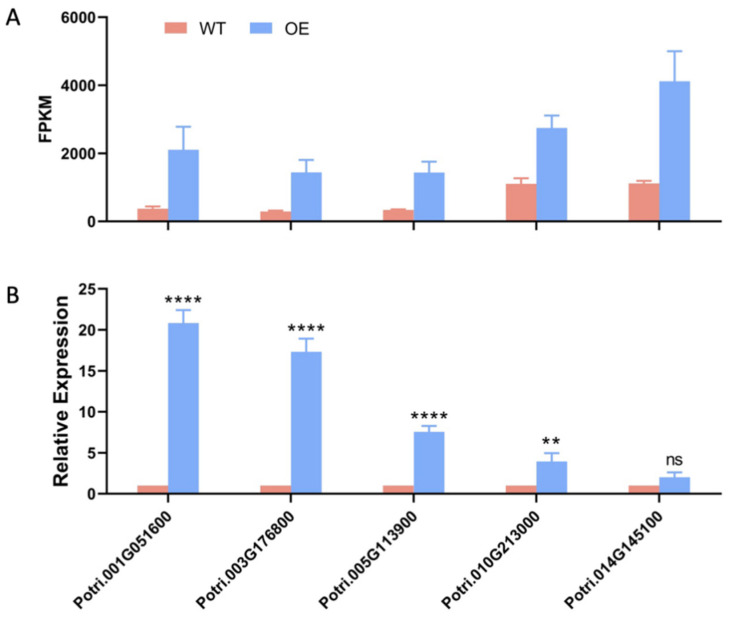
Verification of salt tolerance genes. (**A**) The FPKM value of the selected genes. (**B**) Analysis of the relative expression levels of the selected genes by qRT-PCR. Significant differences are marked with asterisks above the error bar (Student’s *t*-test; ** *p* ≤ 0.01, **** *p* < 0.0001, ns: not significant).

Based on the transcriptome data, it was found that there are up to five genes (Potri.001G051600, Potri.003G176800, Potri.005G113900, Potri.010G213000, and Potri.014G145100) involved in the flavonoid biosynthesis process among the DEGs for which the FPKM (Fragments Per Kilobase of exon model per Million mapped fragments) value was more than 50 in the WT and the up-regulation fold change was greater than 2 (Figure 6A). To investigate whether these genes have played a role in salt stress, an analysis of the expression patterns of these genes was further conducted by qRT-PCR. The results showed that four genes (Potri.001G051600, Potri.003G176800, Potri.005G113900, and Potri.010G213000) were up-regulated in overexpression lines after salt treatment, of which Potri.001G051600 and Potri.003G176800 are chalcone synthases (CHS, EC2.3.1.74) that function as the first key enzyme in flavonoid biosynthesis [35]; Potri.005G113900 is a naringenin 3-dioxygenase (F3H, EC1.14.11.9) playing a crucial role in the production of flavonols and anthocyanins [36], and Potri.010G213000 is a chalcone isomerase (CHI, EC5.5.1.6) that functions as the second rate-limiting enzyme in the biosynthetic pathway of flavonoids [37]. It should be noted that flavonoids are involved in a variety of biological activities in plants and can protect plants from different biological (plant parasitic nematodes, fungi, and bacteria) and abiotic stresses (salt, drought, ultraviolet, and high and low temperatures) [38].

Then, based on the transcriptome, we found all up-regulated genes within the GO:0009813 (flavonoid biosynthetic process from GO analysis) and map00941 (flavonoid biosynthesis from KEGG analysis) and conducted an expression analysis of these genes. A total of 14 genes were identified, including the 5 genes mentioned previously. We specifically focused on two genes predicted to be CHI (Potri.001G051600 and Potri.003G176900), one predicted to be F3H (Potri.005G113700), and one predicted to be DFR (Dihydroflavonol 4-reductase, EC1.1.1.215, Potri.002G033600). Following salt treatment, we performed qRT-PCR analysis on these genes. The results indicated that Potri.001G051600 and Potri.005G113700 were significantly up-regulated in the overexpression lines, while Potri.002G033600 was significantly down-regulated, and Potri.003G176900 showed no significant change (Figure S2). These findings suggest that the predicted CHI and DFR genes are regulated by *PtrLBD41* under salt stress.

The above findings acquired from experiments that we have conducted suggest that the overexpression of the *PtrLBD41* gene can enhance salt tolerance in plants. The *PtrLBD41* gene is involved in the flavonoid biosynthesis pathway by regulating the expression of various genes within the pathway, including the first and the second rate-limiting enzymes in the biosynthetic pathway of flavonoids.

## 3. Discussion

Currently, as the most widely planted economic tree in the world and a model plant among woody plants, *P. trichocarpa* has aroused increasing interest among researchers in recent years. The rising salinity poses a global threat to forestry production and natural ecosystems, and seriously affects the growth, development, and yield of various plants at different stages of their life [39]. Exposure to salt stress would trigger changes in plants at the physiological, biochemical, and molecular levels to resist the damage caused by various environmental stresses [40]. TFs strongly influence plant growth and development and are key regulators of the responses to environmental stress.

LBD genes, a plant-specific TF family, play a key role in lateral organ development, plant regeneration, photomorphogenesis, pathogen response, and microtubule organization differentiation, as well as anthocyanin and nitrogen metabolism [12]. Plant growth is irreversible, and it is easily affected by abiotic stresses such as salt, heavy metals, waterlogging, and drought during the whole growth and development process. Salt is one of the main abiotic stress factors that affect the normal growth and development of plants. In our research, we have isolated and cloned the *PtrLBD41* gene from *P. trichocarpa*, of which the *PtrLBD41* gene contains 891 bases and encodes a hydrophilic protein of 297 amino acids. An analysis of the domains revealed that the N-terminus of PtrLBD41 proteins is highly conserved and contains the LOB domain, like every gene in the LBD TF family. As a class II member, the PtrLBD41 protein contains a zinc finger motif CX2CX6CX3C, unlike class I proteins that contain three conserved domains (CX2CX6CX3C, GAS-block motif, and LX6LX3LX6L). Research shows that BcAS2, a TF containing an LOB domain, is expressed in the nucleus and cytomembrane [41]. TaSDIR1-4A and SiMYBS3 are localized in the plasma membrane and nucleus [42,43]. The Arabidopsis TFs NAC062 and NTL8 are localized on the plasma membrane [44,45]. Likewise, we found that PtrLBD41 is localized in the cell membrane.

The above experiment results showed that the expression of *PtrLBD41* was strongly induced by salt treatment, low temperature, dehydration, and ABA, indicating that such a TF plays a major role in regulating the abiotic stress response network in *P. trichocarpa* (Figure 3). In particular, the LBD gene was up-regulated by more than 174-fold in the salt treatment (Figure 3A). Likewise, the salt treatment caused the level of expression of *SbLBD32* in *Sorghum bicolor* to amplify [46], and overexpressing *PvLBD12* was found to increase salt tolerance in *Panicum virgatum* [29].

We also found that the chlorophyll content of the transgenic lines was significantly higher than that of the WT after salt stress, indicating that the overexpression of *PtrLBD41* protected the plants from more stress to prevent the leaves from curling and yellowing (Figure 4B,C). Chlorophyll is one of the most important pigments in the entire biosphere [47,48], and its degradation can be triggered by exposure to abiotic stresses or naturally occurs during plant senescence, which, in order to counteract the detrimental environment, may manifest in multiple adaptive responses such as leaf decay, and the wilting or yellowing of plants, or directly in the premature end of the life cycle [49,50,51]. The chlorophyll fluorescence parameter Fv /Fm, measured after 20 min of dark adaptation as an indicator of the maximum quantum efficiency of photosystem II (PSII) photochemistry, has been widely used for early stress detection in plants [52]. We also found that the Fv/Fm of the WT decreased significantly after salt treatment and was significantly lower than that of the overexpressed lines, while no significant change was observed in the Fv/Fm of the overexpressed lines compared to the control conditions (Figure 4B,D). The higher chlorophyll content observed in the overexpression lines suggests that the overexpression of *PtrLBD41* enhances the salt tolerance of plants. MDA, a small reactive organic molecule that is ubiquitous in eukaryotes [53], is the main product of plant membrane lipid peroxidation under salt stress, the level of which reflects the degree of cell membrane damage. It is therefore deduced that the MDA content can reflect the salt stress and salt tolerance of plants [54]. Our experiments found that the MDA content of the WT was significantly increased compared with the control group after salt treatment, and the MDA content of WT plants was significantly lower than that in overexpressed *PtrLBD41* plants under salt treatment (Figure 4B,E). Taken together, the above results suggest that *PtrLBD41* functions as a positive regulator in the response to salt stress in *P. trichocarpa*.

To characterize the genes and enrichment pathways affected by the overexpression of *PtrLBD41*, we performed a transcriptome analysis in leaves and analyzed the function and enrichment pathway of DEGs by GO and KEGG. The GO enrichment analysis showed that the DEGs were enriched in the plasma membrane in terms of the cellular component, and cell wall organization in terms of the biological process (Figure 5C). This finding is consistent with the results of cellular membrane localization (Figure 2), as the majority of TFs are typically localized to the nucleus. In order to gain a deeper insight into the role and functioning mechanism of *PtrLBD41* in salt stress, a further analysis of DEGs was conducted, and it is found that five genes in which the FPKM up-regulation exceeded 50-fold in the WT belong to flavonoid biosynthesis pathway (Figure 6A).

Four candidate genes were up-regulated under salt stress (Figure 6B), of which Potri.001G051600 and Potri.003G176800 are CHSs (EC2.3.1.74), Potri.005G113900 is a F3H (EC1.14.11.9), and Potri.010G213000 is a CHI (EC5.5.1.6). CHS is a key enzyme that catalyzes the first step of the flavonoid biosynthesis pathway, is an essential enzyme involved in the production of flavonoid derivatives, and plays an important role in biological processes related to plant growth [55]. It can produce the skeleton of various plant secondary metabolites, including chalcones, stilbenes, phloroglucinols, resorcinols, benzophenones, biphenyls, bibenzyls, chromones, acridones, pyrones, and curcuminoids [56]. The overexpression of EaCHS1 alters the accumulation of flavonoids in plants and regulates the salt stress tolerance [57]. CHI is the second rate-limiting enzyme in the flavonoid biosynthesis pathway. The *MpCHI*, or flavonoid biosynthesis pathway, can regulate the resistance of *M. pinnata* to salt stress [58]. F3Hs catalyzes an early step in the flavonoid pathway, providing precursors for many classes of flavonoid compounds [59]. The overexpression of *CsF3H* increased the salt stress tolerance of tobacco [60]. The results of the above analysis suggest that *PtrLBD41* can enhance the salt stress tolerance in *P. trichocarpa* and is involved in the flavonoid biosynthesis pathway, which plays an important role in plant resistance to salt stress.

This paper provides a new theoretical basis for the research on the resistance of woody plants to salt stress.

## 4. Materials and Methods

### 4.1. Plant Materials and Growth Conditions

All experiments were performed with *P. trichocarpa* genotype Nisqually-1. Tissue culture seedlings were cultured on medium supplemented with 25 g/L sucrose, 5.8 g/L agar, 2.41 g/L wood plant medium (WPM), and 0.1 mg /L indole-3-butyric acid (IBA) in an artificial climate chamber. Soil-culture plants were cultured on a mixed substrate consisting of nutrition soil and vermiculite at a ratio of 2:1. Culture conditions were as follows: 25 °C, 46 μmol photons m^−2^/s, and 16 h light/8 h dark cycles.

### 4.2. Abiotic Stress

Four types of abiotic stresses were applied on 28-day-old tissue-cultured seedlings. Selected uniformly grown tissue-cultured seedlings were cleared of root fixation medium rapidly and placed separately in liquid WPM medium, with one group supplemented with 200 mM NaCl and another with 100 mM ABA, and samples were collected at 0, 1.5, 3, 6, 12, and 24 h for salt and ABA treatments, respectively. The method refers to the literature [61,62]. The tissue-cultured seedlings with the bottom medium removed were placed on clean filter paper to simulate drought, and samples were collected at 0, 0.5, 1, 3, 6, and 12 h for dehydration stress. The method refers to the literature [63]. The seedlings were placed directly into a 4 °C refrigerator for low-temperature stress, and samples were collected at 0, 1.5, 3, 6, 12, and 24 h.

In addition, soil cultures were obtained by transplanting 28-day-old tissue culture seedlings into pots, which were subject to irrigation of 200 mM NaCl for 7 days after 50 days of cultivation in soil, and samples were collected at 0 and 7 days (168 h). Photos were taken for phenotypic analysis.

### 4.3. Bioinformatics Analysis

The primary structure of the PtrLBD41 protein was obtained on ExPASy-ProtParam with ExPASy-ProtScale (http://www.expasy.org/, accessed on 10 July 2024) analysis website. Amino acid sequences of PtrLBD41 protein and the LBD proteins of other plants were collected from NCBI (https://www.ncbi.nlm.nih.gov/, accessed on 10 July 2024). The phylogenetic tree was generated utilizing the neighbor-joining method in MEGA 7. And multiple sequence comparison was performed by DNAMAN.

We have predicted the structural domains of PtrLBD41 protein with the help of SMART (http://smart.embl-heidelberg.de/, accessed on 10 July 2024) and InterPro (https://www.ebi.ac.uk/interpro/, accessed on 10 July 2024) databases, and used SOPMA to predict the secondary structure of PtrLBD41 protein (https://npsa.lyon.inserm.fr/cgi-bin/npsa_automat.pl?page=/NPSA/npsa_sopma.html, accessed on 10 July 2024). For the prediction of its tertiary structure, we have used the SWISS-MODEL (https://swissmodel.expasy.org/, accessed on 10 July 2024) as the protein structure homology modelling server.

### 4.4. Extraction of Total RNA and Cloning of PtrLBD41

Omega E.Z.R.A.^®^ Plant RNA Kit(Omega Biotek Inc., Norcross, GA, USA) was used to extract the total RNA from the harvested *P. trichocarpa* plants, and TOYOBO ReverTra Ace^®^ qPCR RT Master Mix (Toyobo Co., Ltd., Osaka, Japan) was used to reverse-transcribe the total RNA into cDNA. A pair of specific primers was designed (PtrLBD41-F/R; Table S1), and PCR reaction was performed with 2 × GoldStar Best MasterMix with Dye (CWbio, Taizhou, China). Electrophoresis was performed with 1% agarose and specific DNA fragments were recovered and purified from agarose gel with Omega Gel Extraction Kit. Then, we ligated the PCR product to the pMD18-T Vector (Takara, Kusatsu, Japan) and subsequently sent them for sequencing.

### 4.5. Determination of the Subcellular Localization of PtrLBD41

The full-length CDS sequence of *PtrLBD41* was ligated to the GFP gene carrying the CaMV35S promoter. The fusion construct (35S: PtrLBD41: *GFP*) and the empty vector (35S: *GFP*) were integrated into the *Agrobacterium tumefaciens* GV3101 strain, injected into lower epidermal cells of tobacco leaves. Infected tobacco was incubated in the dark for 24 h, followed by normal conditions for 48 h. Tobacco leaves were photographed with a confocal laser scanning microscope (LSM800 with Airyscan, Carl Zeiss, Oberkochen, Germany). Primers are shown in Supplemental Table S1.

### 4.6. RT-qPCR Evaluation

The manufacturer’s instructions for reactions on a 96-well fluorescent qRT-PCR instrument (Roche Light Cycler 480 II, Basel, Switzerland) were followed for conducting the qRT-PCR utilizing UltraSYBR Mixture luminous dye (CWBIO, Taizhou, China). The relative abundance of transcripts was determined using the 2^−ΔΔCT^ method. Primers are shown in Supplemental Table S1.

### 4.7. Construction of P. trichocarpa Overexpressing PtrLBD41 Plants

The fusion construct (35S: PtrLBD41: *GFP*) *Agrobacterium tumefaciens* GV3101 strain was transformed into *P. trichocarpa* stem [64]. Positive plants were examined by qRT-PCR. Primers are shown in Table S1.

### 4.8. Determination of Physiological Indexes

The chlorophyll content and Fv/Fm were measured on living leaves by Chlorophyll Content Meter CCM-200 (OPTI-SCIENCES, Hudson, OH, USA) and Pocket PEA (Hansatech, British, UK) separately. MDA content in harvested soil-cultured leaves was determined by MDA content kit (Grace Biotechnology, Nanjing, China).

### 4.9. RNA Sequencing and Analysis

To perform RNA sequencing (RNA-Seq) analysis, WT and OE4 line transgenic plants were selected at the age of 28 days, and their leaves were collected to generate nine samples; each sample was taken from three plants. RNA-Seq experiments and bioinformatics analyses were carried out by ANOROAD (Beijing, China).

### 4.10. Statistical Analysis

We used GraphPad Prism 8 software for statistical analysis and visualization of the sample data. All the experiments were repeated independently at least three times, and the differences of measured parameters were statistically significant, tested by Student’s *t*-test; there was a significant difference at * *p* ≤ 0.05, ** *p* ≤ 0.01, *** *p* ≤ 0.001, and **** *p* < 0.0001.

## 5. Conclusions

In summary, we have isolated and cloned *PtrLBD41*, an LBD TF family gene responsive to salt, low temperature, dehydration, and ABA treatment, and the PtrLBD41 protein is localized to the cell membrane. The overexpression of *PtrLBD41* in *P. trichocarpa* enhances the resistance to salt stress compared with WT plants. Transcriptome and qRT-PCR analyses have elucidated that PtrLBD41 significantly up-regulates the expression of key flavonoid biosynthetic genes, including CHS, CHI, and F3H. These genes, pivotal to flavonoid production, are up-regulated in response to salt stress and are significantly involved in the flavonoid biosynthesis pathway. These findings have provided new insights into the response mechanisms of *P. trichocarpa* to abiotic stress, hence laying the foundation for further investigations into the relationship between *PtrLBD41* and the flavonoid pathway.

## Data Availability

The data presented in this study are available in the Supplementary Materials.

## Supplementary Materials

The supporting information can be downloaded at: https://www.mdpi.com/article/10.3390/ijms252212349/s1.

## References

[1] Agarwal P.K., Shukla P.S., Gupta K., Jha B. (2013). Bioengineering for salinity tolerance in plants: State of the art. Mol. Biotechnol..

[2] Hazell P., Wood S. (2007). Drivers of change in global agriculture. Philos. Trans. R. Soc. B Biol. Sci..

[3] Qadir M., Quillérou E., Nangia V., Murtaza G., Singh M., Thomas R.J., Drechsel P., Noble A.D. (2014). Economics of salt-induced land degradation and restoration. Nat. Resour. Forum.

[4] Han D., Zhou Z., Du M., Li T., Wu X., Yu J., Zhang P., Yang G. (2020). Overexpression of a *Malus xiaojinensis* WRKY transcription factor gene (*MxWRKY55*) increased iron and high salinity stress tolerance in *Arabidopsis thaliana*. In Vitro Cell. Dev. Biol. Plant.

[5] Ismail A., Takeda S., Nick P. (2014). Life and death under salt stress: Same players, different timing?. J. Exp. Bot..

[6] Yang Y., Guo Y. (2018). Unraveling salt-stress signaling in plants. J. Integr. Plant Biol..

[7] Muchate N.S., Nikalje G.C., Rajurkar N.S., Suprasanna P., Nikam T.D. (2016). Plant Salt Stress: Adaptive Responses, Tolerance Mechanism and Bioengineering for Salt Tolerance. Bot. Rev..

[8] Julkowska M., Testerink C. (2015). Tuning plant signaling and growth to survive salt. Trends Plant Sci..

[9] Wang Z., Wang M., Liu L., Meng F. (2013). Physiological and proteomic responses of diploid and tetraploid black locust (*Robinia pseudoacacia* L.) subjected to salt stress. Int. J. Mol. Sci..

[10] Bazihizina N., Colmer T.D., Cuin T.A., Mancuso S., Shabala S. (2019). Friend or foe? Chloride Patterning in Halophytes. Trends Plant Sci..

[11] Gong Z. (2021). Plant abiotic stress: New insights into the factors that activate and modulate plant responses. J. Integr. Plant Biol..

[12] Xu C., Luo F., Hochholdinger F. (2016). Lob domain proteins: Beyond lateral organ boundaries. Trends Plant Sci..

[13] Yang Y., Yu X., Wu P. (2006). Comparison and evolution analysis of two rice subspecies LATERAL ORGAN BOUNDARIES domain gene family and their evolutionary characterization from *Arabidopsis*. Mol. Phylogenetics Evol..

[14] Chanderbali A.S., He F., Soltis P.S., Soltis D.E. (2015). Out of the Water: Origin and Diversification of the LBD Gene Family. Mol. Biol. Evol..

[15] Matsumura Y., Iwakawa H., Machida Y., Machida C. (2009). Characterization of genes in the ASYMMETRIC LEAVES2/LATERAL ORGAN BOUNDARIES (AS2/LOB) family in *Arabidopsis thaliana*, and functional and molecular comparisons between AS2 and other family members. Plant J..

[16] Iwakawa H., Ueno Y., Semiarti E., Onouchi H., Kojima S., Tsukaya H., Hasebe M., Soma T., Ikezaki M., Machida C. (2002). The *ASYMMETRIC LEAVES2* gene of *Arabidopsis thaliana*, Required for Formation of a Symmetric Fat Leaf Lamina, Encodes a Member of a Novel Family of Proteins Characterized by Cysteine Repeats and a Leucine Zipper. Plant Cell Physiol..

[17] Majer C., Hochholdinger F. (2011). Defining the boundaries: Structure and function of LOB domain proteins. Trends Plant Sci..

[18] Borghi L., Bureau M., Simon R. (2007). *Arabidopsis JAGGED LATERAL ORGANS* Is Expressed in Boundaries and Coordinates *KNOX* and *PIN* Activity. Plant Cell.

[19] Bortiri E., Chuck G., Vollbrecht E., Rocheford T., Martienssen R., Hake S. (2006). *ramosa2* Encodes a LATERAL ORGAN BOUNDARY Domain Protein that Determines the Fate of Stem Cells in Branch Meristems of Maize. Plant Cell.

[20] Liu H., Wang S., Yu X., Yu J., He X., Zhang S., Shou H., Wu P. (2005). ARL1, a LOB-domain protein required for adventitious root formation in rice. Plant J..

[21] Xu L., Xu Y., Dong A., Sun Y., Pi L., Xu Y., Huang H. (2003). Novel *as1* and *as2* defects in leaf adaxial-abaxial polarity reveal the requirement for *ASYMMETRIC LEAVES1* and *2* and *ERECTA* functions in specifying leaf adaxial identity. Development.

[22] Fan M., Xu C., Xu K., Hu Y. (2012). LATERAL ORGAN BOUNDARIES DOMAIN transcription factors direct callus formation in *Arabidopsis regeneration*. Cell Res..

[23] Yordanov Y.S., Regan S., Busov V. (2010). Members of the LATERAL ORGAN BOUNDARIES DOMAIN transcription factor family are involved in the regulation of secondary growth in Populus. Plant Cell.

[24] Lu Q., Shao F., Macmillan C., Wilson I.W., van der Merwe K., Hussey S.G., Myburg A.A., Dong X., Qiu D. (2018). Genomewide analysis of the lateral organ boundaries domain gene family in *Eucalyptus grandis* reveals members that differentially impact secondary growth. Plant Biotechnol. J..

[25] Tuskan G.A., Difazio S., Jansson S., Grigoriev I., Hellsten U., Putnam N., Ralph S., Rombauts S., Salamov A., Schein J. (2006). The Genome of Black Cottonwood, *Populus trichocarpa* (Torr & Gray). Science.

[26] Han Z., Yang T., Guo Y., Cui W.H., Yao L.J., Li G., Wu A.M., Li J.H., Liu L.J. (2021). The transcription factor PagLBD3 contributes to the regulation of secondary growth in *Populus*. J. Exp. Bot..

[27] Yu J., Zhou C., Li D., Li S., Lin Y.C.J., Wang J.P., Chiang V.L., Li W. (2022). A PtrLBD39-mediated transcriptional network regulates tension wood formation in *Populus trichocarpa*. Plant Commun..

[28] Yu J., Gao B., Li D., Li S., Chiang V.L., Li W., Zhou C. (2024). Ectopic Expression of *PtrLBD39* Retarded Primary and Secondary Growth in *Populus trichocarpa*. Int. J. Mol. Sci..

[29] Guan C., Wu B., Ma S., Zhang J., Liu X., Wang H., Zhang J., Gao R., Jiang H., Jia C. (2023). Genome-wide characterization of LBD transcription factors in switchgrass (*Panicum virgatum* L.) and the involvement of *PvLBD12* in salt tolerance. Plant Cell Rep..

[30] Ariel F.D., Diet A., Crespi M., Chan R.L. (2010). The LOB-like transcription factor MtLBD1 controls *Medicago truncatula* root architecture under salt stress. Plant Signal. Behav..

[31] Wu M., He W., Wang L., Zhang X., Wang K., Xiang Y. (2023). PheLBD29, an LBD transcription factor from *Moso bamboo*, causes leaf curvature and enhances tolerance to drought stress in transgenic *Arabidopsis*. J. Plant Physiol..

[32] Feng X., Xiong J., Zhang W., Guan H., Zheng D., Xiong H., Jia L., Hu Y., Zhou H., Wen Y. (2022). *ZmLBD5*, a class-II LBD gene, negatively regulates drought tolerance by impairing abscisic acid synthesis. Plant J..

[33] Xiong J., Zhang W., Zheng D., Xiong H., Feng X., Zhang X., Wang Q., Wu F., Xu J., Lu Y. (2022). *ZmLBD5* increases drought sensitivity by suppressing ROS accumulation in *Arabidopsis*. Plants.

[34] Jiao P., Wei X., Jiang Z., Liu S., Guan S., Ma Y. (2022). ZmLBD2 a maize (*Zea mays* L.) lateral organ boundaries domain (LBD) transcription factor enhances drought tolerance in transgenic *Arabidopsis thaliana*. Front. Plant Sci..

[35] Wang H., Wang W., Zhan J., Yan A., Sun L., Zhang G., Wang X., Ren J., Huang W., Xu H. (2016). The accumulation and localization of chalcone synthase in grapevine (*Vitis vinifera* L.). Plant Physiol. Biochem..

[36] Dai M., Kang X., Wang Y., Huang S., Guo Y., Wang R., Chao N., Liu L. (2022). Functional Characterization of Flavanone 3-Hydroxylase (F3H) and Its Role in Anthocyanin and Flavonoid Biosynthesis in Mulberry. Molecules.

[37] Yin Y.C., Zhang X.D., Gao Z.Q., Hu T., Liu Y. (2019). The Research Progress of Chalcone Isomerase (CHI) in Plants. Mol. Biotechnol..

[38] Zhuang W.B., Li Y.H., Shu X.C., Pu Y.T., Wang X.J., Wang T., Wang Z. (2023). The Classification, Molecular Structure and Biological Biosynthesis of Flavonoids, and Their Roles in Biotic and Abiotic Stresses. Molecules.

[39] Acharya B.R., Gill S.P., Kaundal A., Sandhu D. (2024). Strategies for combating plant salinity stress: The potential of plant growth-promoting microorganisms. Front. Plant Sci..

[40] Zou J., Liu C., Liu A., Zou D., Chen X. (2012). Overexpression of *OsHsp17.0* and *OsHsp23.7* enhances drought and salt tolerance in rice. J. Plant Physiol..

[41] Lin Y., Hou H., Zhang Y., Hou X. (2021). Overexpression of a Pak Choi Gene, *BcAS2*, Causes Leaf Curvature in *Arabidopsis thaliana*. Genes.

[42] Meng Y., Lv Q., Li L., Wang B., Chen L., Yang W., Lei Y., Xie Y., Li X. (2024). E3 ubiquitin ligase TaSDIR1-4A activates membrane-bound transcription factor TaWRKY29 to positively regulate drought resistance. Plant Biotechnol. J..

[43] Liu X., Zhang S., Sun M., Guo Y., Zhao S., Zhou X., Bai X., Dai K., Li H., Yuan X. (2023). *SiMYBS3*, Encoding a *Setaria italica* Heterosis-Related MYB Transcription Factor, Confers Drought Tolerance in *Arabidopsis*. Int. J. Mol. Sci..

[44] Yang Z.T., Lu S.J., Wang M.J., Bi D.L., Sun L., Zhou S.F., Song Z.T., Liu J.X. (2014). A plasma membrane-tethered transcription factor, NAC062/ANAC062/NTL6, mediates the unfolded protein response in *Arabidopsis*. Plant J..

[45] Kim S., Kim S., Park C. (2007). A membrane-associated NAC transcription factor regulates salt-responsive flowering via *FLOWERING LOCUS* T in *Arabidopsis*. Planta.

[46] Wang S., Bai Y., Shen C., Wu Y., Zhang S., Jiang D., Guilfoyle T.J., Chen M., Qi Y. (2010). Auxin-related gene families in abiotic stress response in *Sorghum bicolor*. Funct. Integr. Genom..

[47] Zhang J.L., Shi H. (2013). Physiological and molecular mechanisms of plant salt tolerance. Photosynth. Res..

[48] Hu X., Khan I., Jiao Q., Zada A., Jia T. (2021). Chlorophyllase, a common plant hydrolase enzyme with a long history, is still a puzzle. Genes.

[49] Hörtensteiner S. (2006). Chlorophyll degradation during senescence. Annu. Rev. Plant Biol..

[50] Takamiya K.I., Tsuchiya T., Ohta H. (2000). Degradation pathway(s) of chlorophyll: What has gene cloning revealed?. Trends Plant Sci..

[51] Shi D.Y., Liu Z.X., Jin W.W. (2009). Biosynthesis, catabolism and related signal regulations of plant chlorophyll. Yi Chuan.

[52] Sharma D.K., Andersen S.B., Ottosen C.O., Rosenqvist E. (2015). Wheat cultivars selected for high Fv /Fm under heat stress maintain high photosynthesis, total chlorophyll, stomatal conductance, transpiration and dry matter. Physiol. Plant..

[53] Morales M., Munné-Bosch S. (2019). Malondialdehyde: Facts and Artifacts. Plant Physiol..

[54] Chen G., Zheng D., Feng N., Zhou H., Mu D., Zhao L., Shen X., Rao G., Meng F., Huang A. (2022). Physiological mechanisms of ABA-induced salinity tolerance in leaves and roots of rice. Sci. Rep..

[55] Kong X., Khan A., Li Z., You J., Munsif F., Kang H., Zhou R. (2020). Identification of chalcone synthase genes and their expression patterns reveal pollen abortion in cotton. Saudi J. Biol. Sci..

[56] Abe I., Morita H. (2010). Structure and function of the chalcone synthase superfamily of plant type III polyketide synthases. Nat. Prod. Rep..

[57] Chen L., Guo H., Lin Y., Cheng H. (2015). Chalcone synthase EaCHS1 from *Eupatorium adenophorum* functions in salt stress tolerance in tobacco. Plant Cell Rep..

[58] Wang H., Hu T., Huang J., Lu X., Huang B., Zheng Y. (2013). The Expression of *Millettia pinnata* Chalcone Isomerase in *Saccharomyces cerevisiae* Salt-Sensitive Mutants Enhances Salt-Tolerance. Int. J. Mol. Sci..

[59] Wang Y., Shi Y., Li K., Yang D., Liu N., Zhang L., Zhao L., Zhang X., Liu Y., Gao L. (2021). Roles of the 2-Oxoglutarate-Dependent Dioxygenase Superfamily in the Flavonoid Pathway: A Review of the Functional Diversity of F3H, FNS I, FLS, and LDOX/ANS. Molecules.

[60] Mahajan M., Yadav S.K. (2014). Overexpression of a tea flavanone 3-hydroxylase gene confers tolerance to salt stress and *Alternaria solani* in transgenic tobacco. Plant Mol. Biol..

[61] Lian X., Zhao X., Zhao Q., Wang G., Li Y., Hao Y. (2021). *MdDREB2A* in apple is involved in the regulation of multiple abiotic stress responses. Hortic. Plant J..

[62] Luan X. (2022). Functional Analysis of PtrbZIP53 Gene on Salt Tolerance in *Populus trichocarpa*. Master’s Thesis.

[63] Zhao Q., Hu R.S., Liu D., Liu X., Wang J., Xiang X.H., Li Y.Y. (2020). The AP2 transcription factor *NtERF172* confers drought resistance by modifying *NtCAT*. Plant Biotechnol. J..

[64] Li S., Zhen C., Xu W., Wang C., Cheng Y. (2017). Simple, rapid and efficient transformation of genotype Nisqually-1: A basic tool for the first sequenced model tree. Sci. Rep..

