# Supplementary material for: Overexpression Analysis of PtrLBD41 Suggests Its Involvement in Salt Tolerance and Flavonoid Pathway in Populus trichocarpa
Source: Int J Mol Sci. 2024 Nov 17;25(22):12349. doi: 10.3390/ijms252212349 (PMC11594897; doi:10.3390/ijms252212349)

Figure S1. Characterization of *PtrLBD41*. PCR for the cloning of *PtrLBD41,* the CDS length of *PtrLBD41* is 891bp. M, DNA marker. The DNA marker is 2000bp.

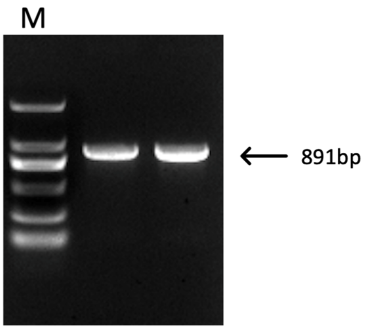

Supplement: Supplementary file 1 [file ijms-25-12349-s001.zip › Figure S1.pdf]
