# Supplementary material for: Overexpression Analysis of PtrLBD41 Suggests Its Involvement in Salt Tolerance and Flavonoid Pathway in Populus trichocarpa
Source: Int J Mol Sci. 2024 Nov 17;25(22):12349. doi: 10.3390/ijms252212349 (PMC11594897; doi:10.3390/ijms252212349)

Figure S2. Expression analysis of Flavonoid pathway genes. (A) The FPKM value of the selected genes. (B) Analysis of the relative expression levels of the selected genes under salt street by qRT-PCR. Significant differences are marked with asterisks above the error bar (Student's t-test; **** $p < 0.0001$, ns: not significant).

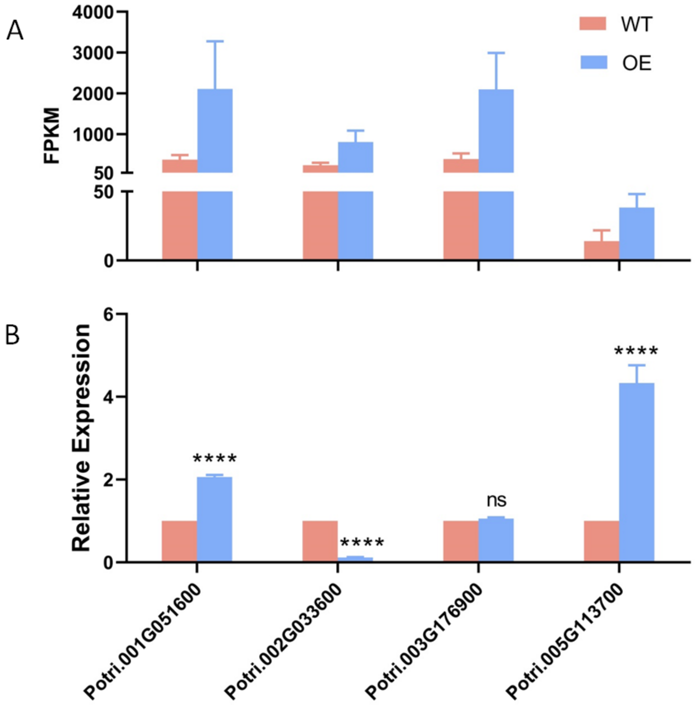

Supplement: Supplementary file 1 [file ijms-25-12349-s001.zip › Figure S2.pdf]
